# Potential application of PS-OCT in the safety assessment of non-steroidal topical creams for atopic dermatitis treatment

**DOI:** 10.1364/BOE.494464

**Published:** 2023-07-14

**Authors:** M. Q. Duan, Robert A. Byers, Simon G. Danby, Sura Sahib, Amy Cha, Chuanbo Zang, John Werth, Roni Adiri, Rosie N. Taylor, Michael J. Cork, Stephen J. Matcher

**Affiliations:** 1 Department of Electronic and Electrical Engineering, The University of Sheffield, The Kroto Building, Broad Lane, Sheffield, S3 7HQ, UK; 2 Sheffield Dermatology Research, Department of Infection and Immunity and Cardiovascular Disease, The University of Sheffield Medical School, Beech Hill Road, Sheffield, S10 2RX, UK; 3 Pfizer, Inc, New York, NY, USA; 4 Pfizer Pharmaceutical Israel LTD, Israel; 5 The Statistical Service Unit, The University of Sheffield, Sheffield, UK

## Abstract

Crisaborole 2% ointment is a non-steroidal treatment for mild-moderate atopic dermatitis (AD) and may produce fewer adverse effects than topical corticosteroids (TCS). We used PS-OCT to quantify dermal collagen at baseline and after 29 days of treatment with crisaborole and betamethasone valerate (BMV), in 32 subjects. PS-OCT detected a mean increase 1 × 10-6, 95% CI (6.3, 1.37) × 10-6 in dermal birefringence following TCS use (p < 0.0001, ad-hoc, not powered), whereas a change of -4 × 10-6, 95% CI (-32, 24) × 10-6 was detected for crisaborole (p = 0.77, ad-hoc, not powered). These results could suggest a differential effect on dermal collagen between the two compounds. PS-OCT may thus find an important role in safety assessment of novel AD treatment’ and larger trials are warranted.

## 1. Introduction

Atopic dermatitis (AD), also known as eczema, is a chronic inflammatory skin disease characterized by pruritus, inflammation, dryness, increased transepidermal water loss (TEWL) and frequent secondary infection of lesion sites [1]. The causes of AD are thought to be both genetic and environmental and the severity of AD symptoms generally correlate with serum IgE levels, indicating that hyper-sensitisation of the immune system is a key factor [2]. Common risk factors which are generally accepted to underlie clinical AD are a dysfunctional skin barrier, usage of some personal wash products, allergy to certain foods and airborne substances and a genetic mutation that alters filaggrin expression in the stratum corneum. The prevalence of AD in most developed countries has increased markedly over the past 7 decades, suggesting a strong role for environmental and lifestyle factors and the condition now affects up to 10-20% of the population [3,4]. Mild AD symptoms can be treated with a regimen of emollient and TCS whereas more severe AD symptoms typically require systematic treatment. It is often reported that patients with AD tend to have a poor quality of life due to constant sleep deprivation and anxiety induced by the continuing pruritus [5]. Psychosocial problems can also arise if a sufferer's visual appearance is strongly affected.

Patients with mild-moderate AD are often prescribed TCS to mitigate AD symptoms. TCS have been popular since the 1950’s due to a combination of high efficacy and low cost. Given the chronic and recurring nature of AD, TCS is often used by patients for long periods. Although the efficacy of TCS is high, long-term use of high potency TCS is not recommended due to common side-effects such as skin atrophy and striae formation [6,7]. Other adverse effects include telangiectasia, senile purpura and stellate pseudoscars, with the skin becoming more fragile over time and its barrier function being compromised. Skin atrophy is frequently associated with skin-aging and TCS-induced skin atrophy can be likened to an accelerated aging process, in some regards.

Hence next-generation therapies, based on non-steroidal compounds, are currently under active development. One such example is crisaborole, a non-steroidal phosphodiesterase 4 (PDE4) inhibitor that has been approved by the FDA in 2016 as a topical anti-inflammatory treatment for patients aged over 2 years of age with mild-moderate AD. Crisaborole blocks the hydrolysis of cyclic adenosine monophosphate (cAMP), thereby degrading the activity of inflammatory cytokines that generate some of the clinical symptoms of AD such as pruritus [8]. Currently, compounds such as crisaborole are more expensive than TCS but are believed to produce fewer adverse effects. A key requirement to demonstrate the cost-effectiveness of alternatives to TCS is thus to demonstrate this in an objective way.

A review study and meta-analysis by Barnes et. al. [9] found that the whole-skin thickness and the epidermal thickness are the most frequently studied parameters, when assessing the atrophogenic activity of TCS. Across 14 clinical studies, which used ultrasound sonography and x-ray radiology to assess whole-skin atrophy after 2-8 weeks of TCS application, whole-skin thickness decreased roughly 5-20%. In comparison, across another 10 clinical studies, which used OCT and histometry to assess epidermal atrophy after 2-6 weeks of TCS application, epidermal thickness decreased roughly 5-40%. Hence epidermal thickness is a sensitive biomarker of TCS-induced atrophy.

Optical Coherence Tomography (OCT) has been investigated as a non-invasive tool to perform longitudinal assessments of TCS-induced epidermal thinning [10]. OCT, often described as an optical equivalent of ultrasound imaging, is a novel, non-invasive tomographic imaging technique that can achieve axial resolutions of 1-10 um, lateral spatial resolutions typically 7 - 20 um [11], FOV typically 4 × 4 mm and 2D imaging speeds ranging from 10’s to 1000’s frames per second. OCT is based on the principle of broad-band interferometry wherein a high-spatial coherence light source of broad emission spectrum (super luminescent diode, supercontinuum source or external cavity tunable laser) illuminates an interferometer, such that the light beam is split into a reference and sample beam. The reference beam is projected onto a reference mirror and the sample beam is directed onto the sample; the two returning beams then interfere on a detector that records the interference pattern as a function of wavelength. Fourier transforming this interference pattern generates information about the depth resolved reflectivity of the sample [12]. Over the past 3 decades, OCT has undergone remarkable technological enhancement, where its’ imaging speed, spatial resolution, image contrast, cost-effectiveness and clinical applicability have all improved [13]. As OCT is capable of imaging tissue with a penetration depth of 1.5-2 mm, skin, as the largest organ in the human body, is particularly suitable for OCT imaging. Since the 1990s, OCT has been successfully applied in dermatology to delineate superficial layers of skin such as epidermis and dermis; identify eccrine sweat glands and observe skin conditions [14]. Standard OCT is thus an ideal tool with which to study epidermal atrophy.

TCS treatment has also been reported to adversely alter collagen metabolism in the dermis, which as a result could also contribute to skin atrophy. Healthy dermal collagen mainly consists of type I and type III fibrillar collagen that are organized in a basket weave-like structure, and a decrease in both types of collagens was reported after TCS treatment [15]. Hence it could be fruitful to use OCT also to study dermal collagen properties and this is the goal of the study reported here.

The OCT variant used in this study, polarization-sensitive OCT (PS-OCT), is a functional extension of OCT where the polarization state of backscattered light is detected, in addition to the light intensity [16]. PS-OCT not only inherits the benefits of standard OCT in terms of speed, depth penetration and resolution, but it can also measure phase retardance, diattenuation and depolarization of tissue. This added contrast allows PS-OCT to differentiate birefringent tissue like muscles, ligament, tendon, and scar tissue from other tissues [17]. In dermatology, PS-OCT has been successfully utilized to image striae [18], identify basal cell carcinoma [19], and assess disease progression in systemic sclerosis [20].

Here we use PS-OCT to study changes in the dermal collagen birefringence caused by TCS administration during a controlled clinical trial. We compare the changes with those caused by crisaborole.

## 2. Method

### 2.1 Subjects and treatment

This study is a part of a larger trial called SMART (Skin bioMARers for atopic eczema Therapy evaluation). SMART is an observer-blind randomized within-subject controlled clinical trial to compare the effects of 4 weeks of TCS use to 4 weeks of crisaborole use. A total of 37 AD patients, within the age range of 18-65 years old (24 female, 14 male) with mixed ethnicity were enrolled, of whom 32 completed the entire study. Crisaborole 2% ointment and betamethasone valerate 0.1% cream (BMV) were applied onto subjects’ inner forearm (in an area free of any clinical signs of eczema) twice per day for 4 weeks. PS-OCT imaging was performed on day 1 (baseline scans - without treatment applied on) and day 29. The SMART study was reviewed by East Midlands – Derby Research Ethics Committee (REC Reference 20/EM/0006), with NCT number – NCT04194814.

### 2.2 PS-OCT system

The system configuration of the in-house PS-OCT system used in this study largely follows Al-Quasi et. al. [21]. The system is built from polarization-maintaining fiber and uses a single, circular input polarization state. It is a swept-source OCT system, based on a commercially available polygon-scanned swept laser (HSL-2000-10-MDL, Santec, Japan), with a center wavelength of 1301 nm and a full-width-half-maximum of 128 nm; the sweep range, sweep rate, duty cycle and output power of such laser are 157 nm, 10kHz, 60% and 20 mW respectively.The system has an axial resolution of roughly 10 µm and a lateral resolutio of roughly 20 µm in air.

**Fig. 1.**
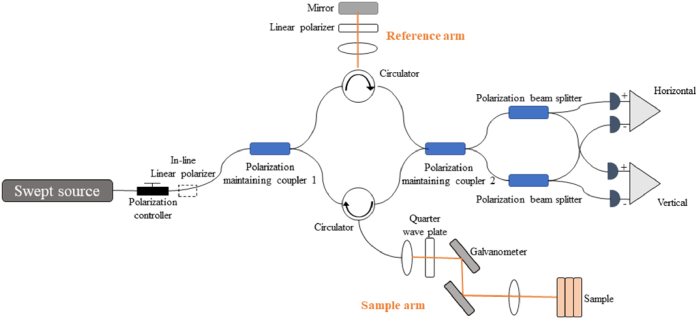
System configuration of our fiber-based PS-OCT.

As shown in Fig. 1, the emission from the fiber-coupled swept source laser initially goes through a polarization controller (PC) and an in-line linear polarizer (IN-LP), whose transmission axis is parallel to the fast axis of the subsequent PM coupler. The light is then split into a reference arm and a sample arm with a ratio of 10:90 by this polarization-maintaining coupler 1 (PMC1, OLCPLP-22-131-10-90-FA, Opto-link Corp., China) and a 3-port polarization-maintaining (PM) circulator (C, OLCIR-P-3-131-300-90-FA, Opto-link Corp., China). The light in the reference arm is collimated and passes through a free-space linear polarizer (LP), whose transmission axis is at 45 degrees to the fast axis of the PM circulator, is reflected by a mirror and then relayed, via the 3rd port of the circulator, to a 50:50 polarization-maintaining coupler, acting as the beam combiner. The fast and slow axes of the combiner are thus illuminated with equal reference intensity. The light in the sample arm is collimated and passed through a quarter wave plate (QWP, NT55-547, Edmund Optics, USA), whose fast axis at 45 degrees to the fast axis of the circulator, thus illuminating the sample with circular polarized light. Volumetric scanning of the sample is performed by an x-y galvanometer pair (Galvo,6215, Cambridge Technology, USA). The reference and sample light interfere at the polarization-maintaining coupler 2 (PMC2, OLCPLP-22-131-50-90-FA, Opto-link Corp., China), producing two signals for balanced detection. The interference signals are decomposed into horizontal and vertical channels by 2 polarizing beam splitters (PBS, PBS-31-P-2-L-3-Q, NovaWave Techno., USA) and detected by 2 balanced detectors (1817-FC, New Focus, USA).

**Fig. 2.**
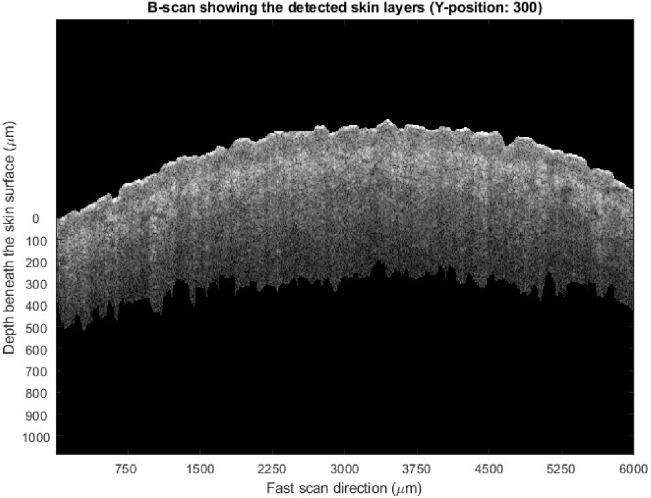
A raw B-scan (Y position of 300 refers to the 300^th^ frame of a volumetric scan) from a volumetric scan of 600 frames before epidermis and dermis segmentation.

**Fig. 3.**
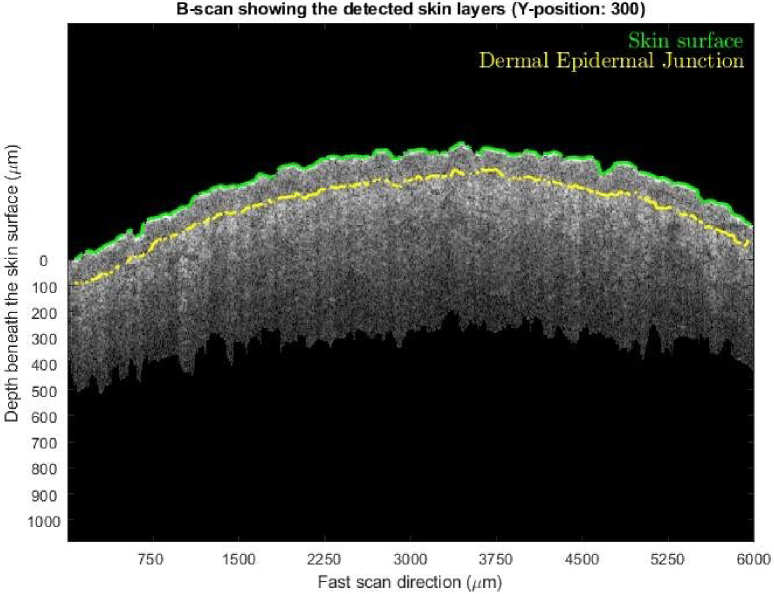
A B-scan (Y position of 300 refers to the 300^th^ frame of a volumetric scan) from a volumetric scan of 600 frames after epidermis and dermis segmentation.

**Fig. 4.**
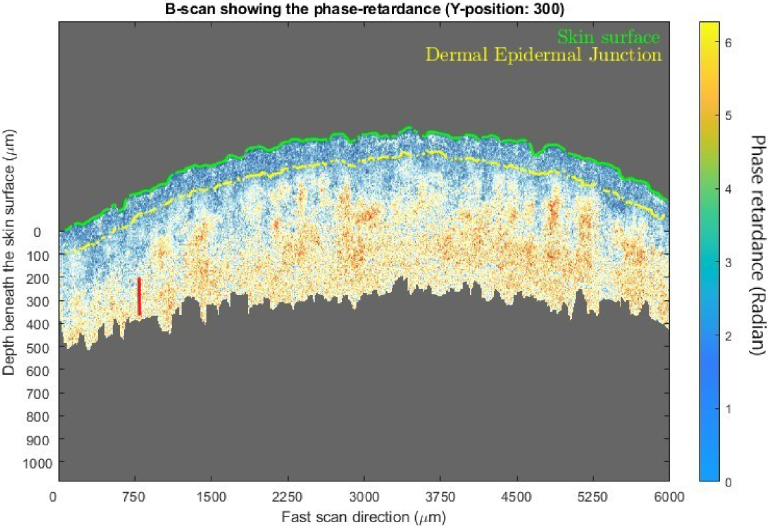
An example B-scan (Y position of 300 refers to the 300^th^ frame of a volumetric scan) from a volumetric scan of 600 frames showing the phase-retardance.

### 2.3 PS-OCT imaging processing

The data processing of the obtained volumetric data sets was done in MATLAB (2019a, MATLAB). Each volumetric data set has a size ( z × *x* × y ) of 512 × 600 × 600. The voxels are roughly 4 × 10 × 10 um size, yielding a volume size of roughly 2 × 6x6 mm. The raw interferometric signals were initially processed (interpolation and inverse FFT) to produce depth resolved complex amplitudes $A_{H}$ & $A_{V}$ in the horizontal and vertical polarization directions. Initially these were utilized to produce B-scans of backscattered intensity via $|A_{H}^{2}\;|\;+\;A_{V}^{2}\;$ . (Fig. 2, Fig. 3) Artifacts in the B-scans such as horizontal stripes from ghost reflections in the optics and vertically displaced ghost surface reflections caused by PM fiber misalignments were removed using a combined wavelet-FFT filtering algorithm previously reported by us [22].

As part of this process, and for subsequent analysis of the dermal birefringence, it was necessary to compute the profiles of the skin surface and dermal-epidermal junction (DEJ). Our semi-automatic algorithm first detects the stratum corneum as a surface edge using sub-pixel edge detection based on the partial area effect [23], finding the edge with the largest positive intensity difference (From dark to bright) along each A-scan and labelling that as the skin surface. The detected surface is compared to a 2D median filtered version of itself (kernel size 40 × 40) and any pixels differing by greater than 10 pixels are assumed to be erroneously detected surface artifacts (e.g., hairs) and interpolated across. Finally, at each lateral location, the surface is locked back onto the nearest detected sub-pixel edge. After the ‘surface boundary’ is detected, the algorithm next detects the DEJ. This junction appears on A-scans as a local-minimum and on B-scans it is shown as a transition from a hypo-reflective epidermis to a more hyper-reflective dermis. This can be detected using a similar method to that of the skin surface, by searching for edges of dark-to-bright transition along each A-scan, however an additional step is added prior to the sub-pixel edge detection. Since non-scattering structures in the dermis (e.g., lymphatics) also display a transition from dark-to-bright along their lower edge, there is an additional source of interference in the DEJ classification. To eliminate these structures, the entire volume is flattened with respect to the surface layer then median filtered in the en face plane. Following this operation, the smaller structures in the DEJ are removed as they are generally narrow in the lateral plane, comparatively the DEJ is persistent across the entire plane and is thus maintained. After this, the same process is used to detect the DEJ as was used for the skin surface. A final manual check is performed by a trained operator to ensure the layer detection was performed accurately, with manual guidance being provided to locally lock the detection of either layer to the correct edges if required.

Quantifying the birefringence of biological tissue by computing the evolution of Stokes vectors on the Poincare sphere is well established and has been reported previously by Götzinger et. al. [24,25]. We have previously used the method to quantify the birefringence of dermal striae [18]. To elaborate, $A_{V}$ and $A_{H}$ are used to calculate the Stokes vector S at each pixel using:

(1) $$\text{S}=\left(\begin{array}{c} \begin{array}{c} \text{I}\\ \text{Q} \end{array}\\ \begin{array}{c} \text{U}\\ \text{V} \end{array} \end{array}\right)=\left(\begin{array}{c} \begin{array}{c} |A_{V}^{2}\;|\;+\;A_{H}^{2}\;.\\ |A_{V}^{2}\;|\;-\;\;A_{H}^{2}\;. \end{array}\\ \begin{array}{c} 2{\text{A}}_{\text{V}}{\text{A}}_{\text{H}}\text{cos}\Delta \theta \\ 2{\text{A}}_{\text{V}}{\text{A}}_{\text{H}}\text{sin}\Delta \theta \end{array} \end{array}\right)$$

Here I , Q , U , and V represent the four elements of the Stokes vector matrix, $|A_{V}^{2}\;|$ and $|A_{H}^{2}\;|$ are the amplitudes of the signals at both detector channels, and Δθ is the phase difference between both channels $\Delta \theta \;=\text{arg}(A_{V})-\text{arg}(A_{H})$ . The Stokes vector is further normalized by dividing Q , U , and V with I and Eq. (1) becomes:

(2) $$\hat{S}=\left(\begin{array}{c} \begin{array}{c} \hat{Q}\\ \hat{U} \end{array}\\ \hat{V} \end{array}\right)\;=\;\left(\begin{array}{c} \begin{array}{c} \text{Q}/\text{I}\\ \text{U}/\text{I} \end{array}\\ V/I \end{array}\right)$$

Following Lin et. al. [18], the phase retardance at a discrete depth $\phi_{r}z_{i}$ is calculated by considering the dot product of the Stokes vector at the skin surface ${\hat{(S}}_{ref})$ and at the discrete depth $z_{i}$ , see Eq. (3).

(3) $${\hat{S}}_{\text{ref}}\cdot {\hat{S}}_{{\text{z}}_{\text{i}}}\;=\;A(z_{i})cos[\left(\phi_{r}(z_{i}\right)]$$

Here, $\phi_{r}(z_{i})$ is an oscillatory term that describes birefringence-induced periodic modulation of the polarization state. $A(z_{i})$ is a more slowly varying envelope, which generally decays and describes the gradual loss of polarization due to scattering, noise and speckle fluctuations. As Lin et. al. noted [18], this equation assumes that the orientation of the fast axis within the target dermal range does not vary strongly with depth.

The phase retardance profile $\phi_{r}(z_{i})$ , which carries the birefringence information, is then extracted by constructing the analytic signal of the LHS and evaluating its phase. The birefringence is estimated from the slope of $\phi_{r}(z_{i})$ with depth, by performing linear regression over the depth range of 200 µm (∼ 50 pixels) beneath the local DEJ.

Because $\phi_{r}(z_{i})$ is wrapped into the primary interval -π to +π radians, $\phi_{r}(z_{i})$ must first be unwrapped in order to accurately measure the slope. To do this, wherever a jump between two neighboring axial pixels exceeds ±π radians, multiples of ±2π are added until the jump is less than π. This is performed using the MATLAB ‘unwrap’ command. The slope *m* is then converted to local birefringence Δ*n* by:

(4) $$\Delta n=m\lambda_{0}\text{RI}/(4\pi \Delta z)$$

Here $\lambda_{0}$ is the center wavelength of the PS-OCT system (1301 nm), Δ*z* is the pixel size in air (4 µm), RI is the refractive index of tissue (1.4).

One problem with such 1D phase unwrapping is that noise fluctuations can cause artifactual phase wraps to be detected and erroneously unwrapped. This causes the phase to increase much faster with depth and an artifactually high birefringence value is then returned. Lin et al. noted this problem when imaging striae [18] and adopted the following mitigation procedure. By examining the histogram and en face parametric plots of the birefringence values, it was established that birefringence values greater than 600 × 10-6 are almost certainly due to noise-induced erroneous phase unwrapping. At such positions, the birefringence is thus re-evaluated using the original, wrapped phase profile instead.

As Lin et. al. [18] noted, the tissue birefringence calculated using this approach should be considered as an ‘effective birefringence’, as the phase profiles are interpreted under the assumption that the collagen orientation (hence birefringence fast axis) remains fixed with depth. Collagen fibers are known to show preferential alignment along lines of mechanical tension in skin (so-called Langer lines) and this alignment becomes more pronounced in striae and scarring. However, collagen organization in normal skin is often described as a “basket weave” structure, wherein bundles of fibers cross each other at nearly 90-degree inclination angle, particularly in younger skin. Still, linear regression of phase retardation with respect to depth has successfully been applied to studying photoaging of skin, in both young and old subjects, by Sakai et al. [26].

## 3. Results and discussion

**Fig. 5.**
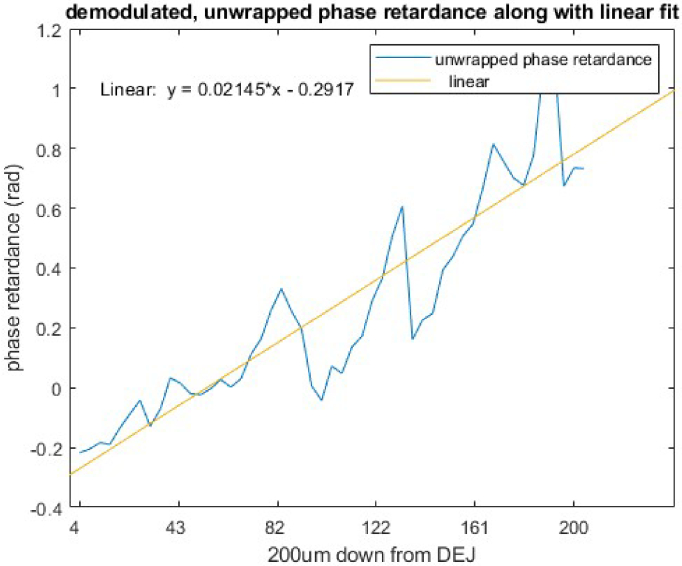
An example of average unwrapped phase retardance over 600 frames at a dermal position (marked in red in Fig. 4.) along with its linear fit.

**Fig. 6.**
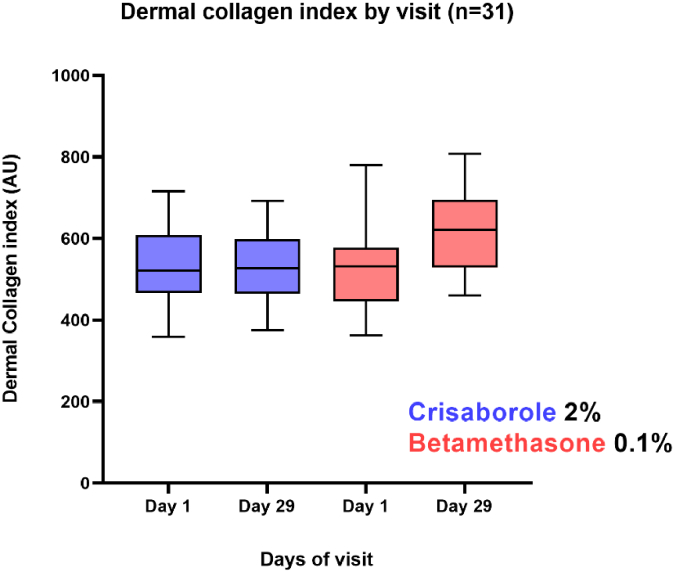
Graph illustration of dermal collagen index change for all subjects under two treatments

32 subjects completed the study and are analyzed herein. PS-OCT scans were performed on day 1 and day 29, with 3 repeats for both arms for each subject, yielding a total of 384 volumetric data sets. A descriptive statistical table of the dermal birefringence values is shown in Table (1). As Table (1) shows, after applying crisaborole the average dermal birefringence value changes from 540 × 10-6 to 536 × 10-6 (n = 32) whereas for BMV the average dermal collagen index increases from 523 × 10-6 to 623 × 10-6 (n = 32). In addition to the descriptive analysis, a paired t-test was performed for all 32 subjects and the results are shown in Table (2) and Fig. 5, Fig. 6. The changes from baseline for two groups were (mean and 95% confidence intervals): -3.99 (-32.32, 24.33) × 10-6 for crisaborole and 100.2 (63.21, 136.84) × 10-6 for BMV. When all subjects underwent 4 weeks of daily application of BMV, their dermal collagen birefringence increased whereas crisaborole 2% did not produce such a change.

**Table 1.** Descriptive statistical table for dermal collagen birefringence for all 32 subjects who completed the study, before (day1) and after (day29) treatment

Metrics	Crisaborole (day1)	BMV (day1)	Crisaborole (day29)	BMV (day29)
25% Percentile	469 × 10-6	451 × 10-6	466 × 10-6	535 × 10-6
Median	521 × 10-6	536 × 10-6	532 × 10-6	626 × 10-6
75% Percentile	612 × 10-6	584 × 10-6	607 × 10-6	694 × 10-6
Mean	540 × 10-6	523 × 10-6	536 × 10-6	623 × 10-6
Standard Deviation	100 × 10-6	91 × 10-6	89 × 10-6	94 × 10-6
Std. Error of mean	17 × 10-6	16 × 10-6	15 × 10-6	16 × 10-6

**Table 2.** Dermal collagen birefringence changes from baseline, for all 32 subjects who completed the study, before (day1) and after (day29) treatment

Metrics	Crisaborole (day29 – day1)	BMV (day29 – day1)
Mean of differences	-3.99 × 10-6	100.02 × 10-6
Standard deviation of differences	74.82 × 10-6	103.3 × 10-6
95% confidence interval	(-32.32 to 24.44) × 10-6	(63.21 to 136.84) × 10-6
P-value (paried T-test)	0.7757	<0.0001

Our finding for TCS (BMV) is in broad agreement with a previous study by Jung et.al. [27], where 16 male subjects were treated on the volar arms with two other TCS compounds, clobetasol propionate and betamethasone dipropionate, for 4 weeks and then compared with a control group. An in-vivo multi-photon microscope was used to measure, among other metrics, a “second-harmonic-to-autofluorescence aging index of dermis” (SAAID). Results showed that, after TCS treatment, there was a significant increase in the SAAID values and a significant increase in the en face multiphoton topography second harmonic generation (MPT-SHG) signal intensities. Such results suggest that the dermal collagen fiber density and/or alignment increased after using TCS, which could be interpreted as dermal fiber reorientation from a random network to a more aligned, or a more compressed structure [27]. An earlier study by Lehmann et.al [28] used electron micron microscopy to evaluate dermal changes caused by the application of TCS. It was reported that 6 weeks of clobetasol propionate treatments caused the fibrous dermal collagen network to reorganize into a more compact structure, which possibly underlies the clinical manifestation of dermal thinning [29].

TCS has been widely used in dermatological therapy for anti-inflammatory purposes since it was introduced in 1952. Despite its effectiveness in reducing inflammatory-related symptoms, adverse effects such as skin atrophy have been reported. While numerous studies reported TCS induces epidermal atrophy and such a parameter dominates the whole skin atrophy, less studies were done focusing on the dermal aspect, it is believed that TCS inhibits collagen synthesis and leads to a loss of dermal extracellular matrix which may explain skin atrophy [9].

The results of this study suggest that there was a structural change in the dermal collagen after using TCS, which was not produced to the same extent when using crisaborole. As noted in the methodology section, the algorithm used in this study quantifies an effective dermal birefringence, by assuming that the birefringence fast axis orientation does not alter strongly with depth. It should be noted that the collagen and elastin fibers in healthy human dermis have been reported to possess a woven mesh-like structure, with a fast axis orientation that changes with depth according to some recent PS-OCT studies [30,31]. Such considerations warrant further investigation and could potentially increase the range of anatomical changes that are consistent with our PS-OCT data. Nonetheless, it remains the case that an alteration in dermal birefringence index, relative to baseline, is observed when subjects treat their skin with TCS whereas, for crisaborole, the change relative to baseline is much smaller and not statistically significant.

### 3.1 Conclusion and future work

We present a novel application of PS-OCT, to assess TCS induced skin atrophy via changes in dermal collagen birefringence rather than epidermal thinning and to compare this with a non-steroidal alternative. While epidermal atrophy is widely reported as a side effect of TCS usage, fewer studies have focused on the effects on dermal collagen. Our detection of an increase in the effective dermal collagen birefringence after application of TCS is consistent with dermal collagen becoming more compacted as result of dermal thinning, although other interpretations may also be compatible with our data. We further find that a non-steroidal alternative to TCS, crisaborole, showed a much smaller change in effective dermal collagen birefringence relative to baseline. Regardless of the precise interpretation that is placed on our effective birefringence values, this observation may provide further evidence that such non-steroidal creams pose less risk of skin atrophy during prolonged atopic dermatitis treatment. To the best of our knowledge, studies reported using PS-OCT to evaluate the effect of anti-inflammatory skin creams on dermal collagen birefringence are limited. PS-OCT may potentially play an important role in assessing the safety of novel topical atopic dermatitis treatments, relative to TCS, and more studies in this area are warranted.

## Data Availability

The data presented in the results are not publicly available at the moment but may be provided by the corresponding author upon reasonable request.
